# Postoperative Recurrence in Allergic Fungal Rhinosinusitis: A Single Center Experience

**DOI:** 10.7759/cureus.81711

**Published:** 2025-04-04

**Authors:** Lenah K AlFadhel, Yazieed Albarrak, Mazen S AlFozan, Tariq Tatwani

**Affiliations:** 1 Otolaryngology - Head and Neck Surgery, Prince Sultan Military Medical City, Riyadh, SAU

**Keywords:** allergic fungal rhinosinusitis, functional endoscopic sinus surgery, postoperative recurrence, rhinosinusitis, surgical complications

## Abstract

Background

Allergic fungal rhinosinusitis (AFRS) is a chronic inflammatory condition of the sinonasal mucosa with a propensity for postoperative recurrence despite surgical intervention. Understanding the factors contributing to recurrence is crucial for optimizing treatment outcomes.

Study aim

This retrospective cohort study aimed to assess demographic, clinical, pathological, and procedural factors associated with postoperative recurrence in AFRS patients undergoing functional endoscopic sinus surgery (FESS).

Methodology

Medical charts of 76 AFRS patients who underwent FESS between January 2008 and August 2021 at Prince Sultan Military Medical City in Riyadh, Saudi Arabia, were reviewed. Demographic data, clinical characteristics, comorbidities, surgical complications, histopathological findings, and postoperative outcomes were analyzed. Statistical analysis included the chi-square test and t-test.

Results

Relapse was observed in 42 (55.3%) patients. Significant predictors of relapse included younger age (24 ± 9 years in relapsing patients vs. 37 ± 17 years in non-relapsing patients, p < 0.001*), shorter time from presentation to surgery (229.8 ± 303.3 days in relapsing patients vs. 883.9 ± 1145.9 days in non-relapsing patients, p = 0.001*), and certain presenting symptoms such as facial swelling (p < 0.001*). Positive intraoperative cultures were more common in relapsing patients (73.3% vs. 36.4%, p = 0.029), and orbital extension was significantly associated with relapse (73.1% vs. 26.9%, p = 0.024). Surgical complications were more frequent among relapsing patients, although not statistically significant (85.7% vs. 52.2%, p = 0.089).

Conclusions

Our study highlights the multifactorial nature of postoperative recurrence in AFRS. Younger age, shorter time from presentation to surgery, specific severe presenting symptoms, positive intraoperative cultures, and orbital extension are significantly associated with higher relapse rates in AFRS patients. These findings highlight the need for tailored management strategies to reduce relapse rates, including aggressive and prolonged medical therapy, comprehensive preoperative assessments, and meticulous surgical and postoperative care.

## Introduction

Allergic fungal rhinosinusitis (AFRS) is a complex inflammatory condition characterized by hypersensitivity reaction to fungal antigens within the sinonasal mucosa [1]. This condition represents a significant challenge in the field of rhinology due to its diverse clinical presentations, chronicity, and potential for recurrence following surgical intervention. Understanding the pathophysiology, clinical manifestations, and treatment outcomes of AFRS is crucial for optimizing patient management and improving long-term outcomes [2,3].

The pathogenesis of AFRS is multifactorial, involving interactions between fungal antigens, host immune responses, and environmental factors. Fungi such as *Aspergillus *species are commonly implicated in AFRS, with the release of fungal proteins and allergens triggering an exaggerated immune response in susceptible individuals [2,4]. This immune dysregulation leads to the production of IgE antibodies, recruitment of eosinophils, and activation of T-helper 2 (Th2) cells, resulting in chronic inflammation and tissue remodeling within the sinonasal cavities [1,2,5].

Clinically, AFRS presents with a spectrum of symptoms ranging from nasal congestion, rhinorrhea, and facial pain to more severe manifestations such as nasal polyps, anosmia, and proptosis. These symptoms often mimic those of other sinonasal disorders, making accurate diagnosis challenging [4,6]. Many risk factors have been linked to fungal sinusitis, such as diabetes mellitus, immunocompromised individuals, and genetic susceptibility. Diagnostic criteria for AFRS, as proposed by Bent and Kuhn [7], include characteristic radiological findings (e.g., opacified sinuses with allergic mucin), positive fungal cultures or histopathology demonstrating eosinophilic mucin, and evidence of type I hypersensitivity to fungal antigens. Sinus opacification could represent multiple pathologies, including chronic rhinosinusitis with or without polyps, fungal ball, AFRS, benign masses, or malignancy [5,6,8].

The management of AFRS typically involves a multidisciplinary approach encompassing medical therapy, surgical intervention, and long-term follow-up [9]. Medical management aims to control inflammation and fungal colonization through the use of intranasal corticosteroids, antihistamines, and antifungal agents [5,9]. Systemic corticosteroids may be indicated for acute exacerbations or severe cases. Intranasal corticosteroids can be used as maintenance therapy after surgical intervention to help control local inflammatory events and keep the sinus outflow patent. The dates in regards to the use of oral and topical antifungal agents are still not clear and not recommended by the International Consensus of Allergy and Rhinology. However, medical therapy alone may not achieve long-term remission, especially in cases of extensive disease or refractory symptoms [10-12].

Functional endoscopic sinus surgery (FESS) remains the cornerstone of surgical management for AFRS. FESS allows for targeted clearance of diseased mucosa, polyps, and fungal debris, thereby improving sinus ventilation and facilitating topical therapies [3,6]. Surgical goals include achieving adequate sinus drainage, reducing fungal load, and restoring normal sinonasal anatomy. Despite the benefits of FESS, recurrence rates in AFRS remain a significant concern, highlighting the need for comprehensive preoperative assessment and postoperative surveillance strategies [10,13].

The factors contributing to postoperative recurrence in AFRS are multifaceted and not fully understood. Potential contributors include incomplete surgical clearance, persistent fungal colonization, immune dysregulation, anatomical variations, and comorbid conditions such as asthma or immunodeficiency [5,6,13]. Identifying predictors of recurrence and optimizing treatment protocols are ongoing areas of research in the management of AFRS [10-12].

Recent advancements in imaging modalities, such as high-resolution computed tomography (CT) and magnetic resonance imaging (MRI), have improved preoperative planning and disease characterization in AFRS. Imaging findings, such as the Lund-Mackay score, can help assess disease severity, guide surgical decision-making, and monitor postoperative outcomes [2,4]. However, the correlation between radiological findings and clinical outcomes, particularly regarding recurrence risk, requires further investigation [10,14,15]. Overall, AFRS represents a complex interplay between fungal pathogens, host immune responses, and environmental factors. While FESS remains an effective treatment modality, challenges such as postoperative recurrence necessitate ongoing research to optimize diagnostic criteria, refine surgical techniques, and develop personalized management strategies for patients with AFRS.

This study aimed to investigate the factors contributing to postoperative recurrence in patients with AFRS following FESS. The objectives included: (i) evaluating the demographic and clinical characteristics of patients diagnosed with AFRS; (ii) assessing the impact of surgical complications on postoperative recurrence in AFRS patients; (iii) analyzing histopathological features and intraoperative culture results in relation to recurrence rates; and (iv) investigating the association between anatomical extensions of the disease (orbital and skull base involvement) and postoperative recurrence in AFRS

## Materials and methods

Study design

This retrospective cohort study aimed to investigate postoperative recurrence in patients with AFRS who underwent FESS. The study design involved the retrospective analysis of patient data from electronic medical records over a specified period.

Study setting

The study was conducted at Prince Sultan Military Medical City in Riyadh, Saudi Arabia. This tertiary care center is equipped with advanced facilities for the diagnosis and management of sinus and nasal disorders, making it a suitable setting for this investigation.

Study population

The study population comprised patients diagnosed with AFRS who underwent FESS between January 2008 and August 2021. A total of 76 patients met the inclusion criteria and were included in the analysis.

Inclusion criteria

Patients were included if they fulfilled the Bent and Kuhn criteria for the diagnosis of AFRS, had undergone FESS during the specified study period, and had complete medical records available for review.

Exclusion criteria

Patients were excluded if they did not meet the criteria for AFRS diagnosis, had incomplete medical records, or had undergone surgical interventions other than FESS for sinus pathology.

Data collection sheet

A structured data collection sheet was used to extract relevant information from electronic medical records. The data collection sheet included demographic details such as age, gender, and ethnicity, clinical characteristics such as comorbidities (e.g., asthma, diabetes mellitus), presenting symptoms, surgical complications, histopathological findings, intraoperative culture results, and postoperative outcomes.

Data management and analysis

Data extracted from the medical records were entered into a spreadsheet for analysis. Statistical analysis was performed using the IBM SPSS Statistics software version 26 (IBM Corp., Armonk, United States). Descriptive statistics were used to summarize demographic and clinical characteristics. Categorical variables were analyzed using chi-square tests, and continuous variables were analyzed using t-tests. Significance levels were set at p < 0.05*.

Ethical considerations

Ethical approval was obtained from the Institutional Review Board (IRB) of Prince Sultan Military Medical City (approval number E-18-3594) before the commencement of the study. Patient confidentiality and data privacy were strictly maintained throughout the study. The study adhered to the principles outlined in the Declaration of Helsinki regarding the ethical conduct of research involving human subjects.

## Results

The study comprised 76 patients diagnosed with AFRS who underwent FESS at Prince Sultan Military Medical City. The age distribution showed that a substantial proportion of patients, 33 (43.4%), were between 18 and 29 years old. This was followed by 20 (26.3%) patients aged 30 to 39, 12 (15.8%) patients aged 40 or older, and 11 (14.5%) patients between nine and 17 years old.

Regarding gender, the majority of the patients were female, constituting 48 (63.2%) members of the cohort, whereas male patients comprised 28 (36.8%). All patients were of Saudi ethnicity, with the majority, 55 (72.4%), residing in the Central region. Other regions included the North with six (7.9%) patients, the South with 12 (15.8%) patients, and the West with three (3.9%) patients.

A notable 48 (63.2%) patients did not have asthma, while 28 (36.8%) were asthmatic. An overwhelming majority, 75 (98.7%), had no nonsteroidal anti-inflammatory drug (NSAID) allergy, with only one (1.3%) patient reporting such an allergy. Diabetes mellitus was present in 10 (13.2%) patients, with six (60%) of these diabetic patients on insulin, three (30%) on oral hypoglycemic agents (OHA), and one (10%) on both treatments.

Family history of similar conditions was reported in 11 (14.5%) patients, while 65 (85.5%) did not have such a history. Allergic history was noted in 12 (15.8%) patients. Smoking was relatively rare, with only three (3.9%) patients being smokers.

In terms of presenting symptoms, nasal obstruction was the most common symptom, experienced by 72 (94.7%) patients. Other frequent symptoms included nasal discharge in 55 (72.4%) patients, headache in 42 (55.3%) patients, anosmia in 36 (47.4%) patients, and facial pressure in 33 (43.4%) patients. Less common symptoms included proptosis in 14 (18.4%) patients, facial swelling in 10 (13.2%), decreased vision in eight (10.5%), epistaxis in seven (9.2%), diplopia in five (6.6%), and postnasal drip (PND) in 28 (36.8%).

The duration of the condition was predominantly chronic, lasting more than four weeks in 73 (96.1%) patients, while it was acute (less than four weeks) in only three (3.9%) patients. The time from presentation to surgery varied, with 42 (55.3%) patients undergoing surgery after more than seven months from the initial presentation. For others, surgery occurred between three to six months in 12 (15.8%) patients, two weeks to two months in 11 (14.5%) patients, and within one week in 11 (14.5%) patients. Table 1 shows the characteristics of the included patients and disease presentation.

**Table 1:** Characteristics of the included patients and disease presentation (n=76) NSAID: nonsteroidal anti-inflammatory drug; PND: postnasal drip

Parameter		Frequency (%)
Age (years)	18 to 29	33 (43.4%)
	30 to 39	20 (26.3%)
	40 or more	12 (15.8%)
	9 to 17	11 (14.5%)
Gender	Female	48 (63.2%)
	Male	28 (36.8%)
Ethnicity	Saudi	76 (100%)
Region	Central	55 (72.4%)
	North	6 (7.9%)
	South	12 (15.8%)
	West	3 (3.9%)
Asthma	No	48 (63.2%)
	Yes	28 (36.8%)
NSAID allergy	No	75 (98.7%)
	Yes	1 (1.3%)
Diabetes mellitus	No	66 (86.8%)
	Yes	10 (13.2%)
Diabetic treatment	Both	1 (10%)
	Insulin	6 (60%)
	Oral antihyperglycemic	3 (30%)
Family history	No	65 (85.5%)
	Yes	11 (14.5%)
History of allergy	No	64 (84.2%)
	Yes	12 (15.8%)
Smoking	No	73 (96.1%)
	Yes	3 (3.9%)
Presenting symptoms	Facial swelling	10 (13.2%)
	Anosmia	36 (47.4%)
	Decreased vision	8 (10.5%)
	Diplopia	5 (6.6%)
	Epistaxis	7 (9.2%)
	Facial pressure	33 (43.4%)
	Headache	42 (55.3%)
	Nasal discharge	55 (72.4%)
	Nasal obstruction	72 (94.7%)
	PND	28 (36.8%)
	Proptosis	14 (18.4%)
Duration of condition	Acute < 4 weeks	3 (3.9%)
	Chronic > 4 weeks	73 (96.1%)
Time from presentation to surgery	1 week or less	11 (14.5%)
	2 weeks to 2 months	11 (14.5%)
	3 months to 6 months	12 (15.8%)
	7 months or more	42 (55.3%)

Among the 76 patients, surgery complications were rare, occurring in only seven (9.2%) patients. The complications included postoperative bleeding in four (57.1%) patients, synechia in three (42.9%) patients, and both cerebrospinal fluid (CSF) leak and intraoperative bleeding each in one (14.3%) patient.

Relapse of AFRS was noted in 42 (55.3%) patients, with 16 (38.1%) of these relapses occurring within 12 months and 26 (61.9%) occurring after 12 months. Hemoglobin A1c (HbA1c) levels were high in 11 (23.4%) patients and low in 36 (76.6%).

Histopathological examination revealed allergic mucin in 20 (26.3%) patients, allergic nasal polyp in 33 (43.4%) patients, fungal hyphae structure in 33 (43.4%) patients, and mucus with inflammatory cells or inflammation in 32 (42.1%) patients. Positive fungal stain was observed in 29 (38.2%) patients. Intraoperative cultures were conducted in 41 (53.9%) patients, yielding positive results in 30 (73.2%) and negative results in 11 (26.8%). *Aspergillus flavus* was the most commonly isolated organism, found in 19 (63.3%) patients with positive cultures.

Preoperative medications included nasal saline in 56 (73.7%) patients, nasal steroid puffs in 52 (68.4%), oral steroids in 32 (42.1%), and antibiotics in 11 (14.5%). Postoperative medications were similar, with nasal saline used by 66 (86.8%) patients, nasal steroid irrigation (Plumicort) by 40 (52.6%), and oral steroids by 37 (48.7%).

Orbital extension of the disease was observed in 26 (34.2%) patients, with 14 (53.8%) of these cases involving the left side. Skull base affection was present in nine (11.8%) patients, evenly distributed between left, right, and both sides. Disease involvement was bilateral in 35 (46.1%) patients, with the left side involved in 20 (26.3%) patients and the right side in 21 (27.6%) patients. Table 2 shows the pathology and outcomes of the disease among the included patients.

**Table 2:** Pathology and outcomes of the disease among the included patients (n=76) HbA1c: hemoglobin A1c; CSF: cerebrospinal fluid

Parameter		Frequency (%)
Surgery complications	No	69 (90.8%)
	Yes	7 (9.2%)
If yes, what are the complications (n=7)	CSF leak	1 (14.3%)
	Intraoperative bleeding	1 (14.3%)
	Postoperative bleeding	4 (57.1%)
	Synechia	3 (42.9%)
Relapse	No	34 (44.7%)
	Yes	42 (55.3%)
Time of relapse (n=42)	Relapse within 12 months	16 (38.1%)
	Relapse in > 12 months	26 (61.9%)
HbA1c	High	11 (23.4%)
	Low	36 (76.6%)
Histopathology	Allergic mucin	20 (26.3%)
	Allergic nasal polyp	33 (43.4%)
	Fungal hyphae structure	33 (43.4%)
	Mucus with inflammatory cells/inflammation	32 (42.1%)
	Not available	5 (6.6%)
	Positive fungal stain	29 (38.2%)
Intraoperative culture	Done	41 (53.9%)
	Not done	35 (46.1%)
Intraoperative culture result (n=41)	Negative	11 (26.8%)
	Positive	30 (73.2%)
Positive culture findings (n=30)	Aspergillus flavus	19 (63.3%)
	Aspergillus fumigatus	1 (3.3%)
	Aspergillus niger	2 (6.7%)
	Aspergillus terreus	1 (3.3%)
	*Bipolaris*/*Drechslera*	4 (13.3%)
	Candida albicans	1 (3.3%)
	Dematiaceous fungus *Bipolaris*	1 (3.3%)
Preoperative medications	Antibiotic	11 (14.5%)
	Antihistamine	19 (25%)
	Intravenous steroids	1 (1.3%)
	Nasal saline	56 (73.7%)
	Nasal steroid irrigation (Plumicort)	14 (18.4%)
	Nasal steroid puffs	52 (68.4%)
	Oral steroids	32 (42.1%)
Postoperative medications	Antibiotics	20 (26.3%)
	Antihistamine	8 (10.5%)
	Nasal saline	66 (86.8%)
	Nasal steroid irrigation (Plumicort)	40 (52.6%)
	Nasal steroid puffs	24 (31.6%)
	Oral steroids	37 (48.7%)
	Xylometazoline	1 (1.3%)
Orbital extension	No	50 (65.8%)
	Yes	26 (34.2%)
Side of orbital extension (n=26)	Both sides	2 (7.7%)
	Left	14 (53.8%)
	Right	10 (38.5%)
Skull base affection	No	67 (88.2%)
	Yes	9 (11.8%)
Side of skull base affection	Both	3 (33.3%)
	Left	3 (33.3%)
	Right	3 (33.3%)
Disease involvement	Both sides	35 (46.1%)
	Left	20 (26.3%)
	Right	21 (27.6%)

**Figure 1:**
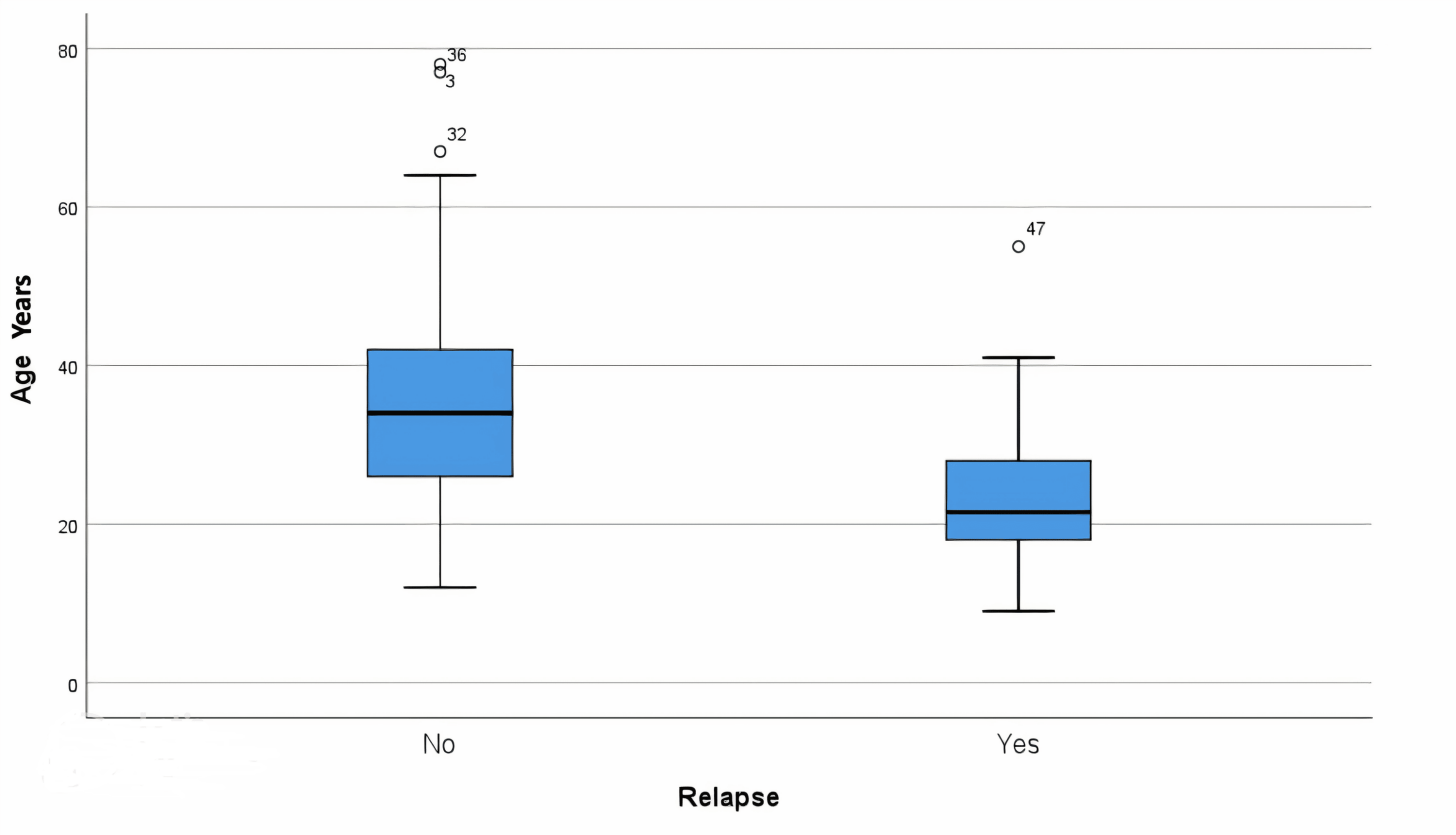
A boxplot of the distribution of age among relapsed and non-relapsed patients A significant association was noted in non-relapsing patients who had a longer mean time to surgery from the time of presentation.

On examining the association between numerical variables and the incidence of relapse, significant differences were observed. The mean age of the patients was 30 years (± 15 years). When stratified by relapse status, non-relapsing patients had a mean age of 37 years (± 17 years), significantly higher than the mean age of 24 years (± 9 years) in relapsing patients (t = -4.335, p = 0.000*). This age distribution is illustrated in Figure 1, where a boxplot highlights the distinct age difference between the two groups.

**Figure 2:**
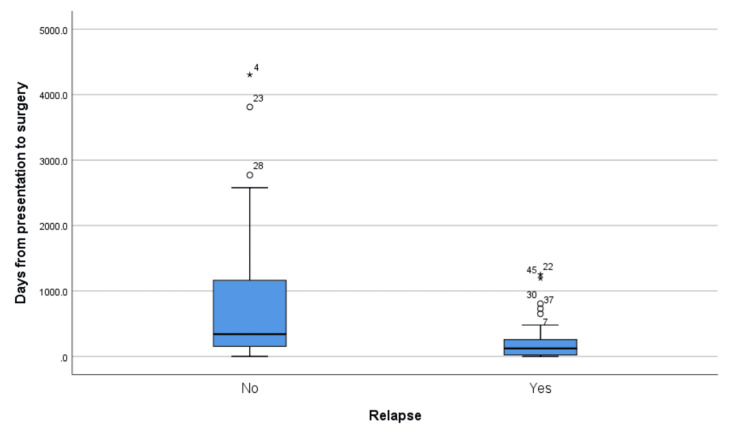
A boxplot of the distribution of days from presentation to surgery among relapsed and non-relapsed patients The interval from presentation to surgery demonstrated a significant association with non-relapsing patients who had a longer mean time to surgery.

The interval from presentation to surgery also demonstrated a significant association with relapse. On average, patients had surgery 522.4 days (± 857.5 days) after presentation. Non-relapsing patients had a longer mean time to surgery, averaging 883.9 days (± 1145.9 days), compared to relapsing patients who had surgery after an average of 229.8 days (± 303.3 days) (t = -3.554, p = 0.001*). Figure 2 depicts this difference, showing a wider range and higher median time to surgery for non-relapsing patients.

The Lund-Mackay score, a measure of disease severity on a scale, averaged 13.87 (± 6.78) across all patients. Non-relapsing patients had a mean score of 12.85 (± 7.86), while relapsing patients had a slightly higher mean score of 14.69 (± 5.73). However, this difference was not statistically significant (t = 1.178, p = 0.243). Table 3 shows the numerical variables and association with relapse among the included patients.

**Table 3:** Numerical variables and association with relapse among the included patients (n=76) (*) indicates a significant p-value < 0.05.

Parameter	Total (n=76)	Non-relapsing (n=34)	Relapsing (n=42)	t	p-value
Age (years)	30 ± 15	37 ± 17	24 ± 9	-4.335	0.000*
Days from presentation to surgery	522.4 ± 857.5	883.9 ± 1145.9	229.8 ± 303.3	-3.554	0.001*
Lund-Mackay score	13.87 ± 6.78	12.85 ± 7.86	14.69 ± 5.73	1.178	0.243

Table 4 provides a detailed breakdown of relapse in relation to various patient characteristics and disease presentations. Age showed a significant correlation with relapse (χ² = 17.8, p = 0.000*). Patients aged 18 to 29 years and nine to 17 years exhibited higher relapse rates of 72.7% and 81.8%, respectively. In contrast, older patients (30 to 39 years) had a lower relapse rate of 35%, and those aged 40 or more had the lowest relapse rate at 16.7%.

**Table 4:** Relapse in association with characters and disease presentation among the included patients (n=76) (*) indicates a significant p-value < 0.05. NSAID: nonsteroidal anti-inflammatory drug; PND: postnasal drip

Parameter		Relapse		χ²	p-value
		Non-relapsing	Relapsing		
Age (years)	18 to 29	9 (27.3%)	24 (72.7%)	17.8	0.000*
	30 to 39	13 (65%)	7 (35%)		
	40 or more	10 (83.3%)	2 (16.7%)		
	9 to 17	2 (18.2%)	9 (81.8%)		
Gender	Female	23 (47.9%)	25 (52.1%)	0.5	0.465
	Male	11 (39.3%)	17 (60.7%)		
Region	Central	26 (47.3%)	29 (52.7%)	0.7	0.882
	North	2 (33.3%)	4 (66.7%)		
	South	5 (41.7%)	7 (58.3%)		
	West	1 (33.3%)	2 (66.7%)		
Asthma	No	21 (43.8%)	27 (56.3%)	0.1	0.821
	Yes	13 (46.4%)	15 (53.6%)		
NSAID allergy	No	34 (45.3%)	41 (54.7%)	0.8	0.365
	Yes	0 (0%)	1 (100%)		
Diabetes mellitus	No	28 (42.4%)	38 (57.6%)	1.1	0.298
	Yes	6 (60%)	4 (40%)		
Diabetic treatment	Both	1 (100%)	0 (0%)	1.7	0.435
	Insulin	4 (66.7%)	2 (33.3%)		
	Oral antihyperglycemic drugs	1 (33.3%)	2 (66.7%)		
Family history	No	30 (46.2%)	35 (53.8%)	0.4	0.546
	Yes	4 (36.4%)	7 (63.6%)		
History of allergy	No	29 (45.3%)	35 (54.7%)	0.1	0.816
	Yes	5 (41.7%)	7 (58.3%)		
Smoking	No	33 (45.2%)	40 (54.8%)	0.2	0.685
	Yes	1 (33.3%)	2 (66.7%)		
Presenting symptoms	Facial swelling	0 (0%)	10 (100%)	36.1	0.000*
	Anosmia	11 (30.6%)	25 (69.4%)		
	Decreased vision	0 (0%)	8 (100%)		
	Diplopia	1 (20%)	4 (80%)		
	Epistaxis	6 (85.7%)	1 (14.3%)		
	Facial pressure	13 (39.4%)	20 (60.6%)		
	Headache	19 (45.2%)	23 (54.8%)		
	Nasal discharge	22 (40%)	33 (60%)		
	Nasal obstruction	31 (43.1%)	41 (56.9%)		
	PND	16 (57.1%)	12 (42.9%)		
	Proptosis	5 (35.7%)	9 (64.3%)		
Duration of condition	Acute < 4 weeks	1 (33.3%)	2 (66.7%)	0.2	0.685
	Chronic > 4 weeks	33 (45.2%)	40 (54.8%)		
Time from presentation to surgery	1 week or less	3 (27.3%)	8 (72.7%)	8.3	0.040*
	2 weeks to 2 months	3 (27.3%)	8 (72.7%)		
	3 months to 6 months	3 (25%)	9 (75%)		
	7 months or more	25 (59.5%)	17 (40.5%)		

Gender did not significantly influence relapse rates, with female patients showing a 52.1% relapse rate and male patients showing a 60.7% relapse rate (χ² = 0.5, p = 0.465). Similarly, the region of residence did not significantly affect relapse rates (χ² = 0.7, p = 0.882).

Asthma status, NSAID allergy, diabetes mellitus, and family history also showed no significant associations with relapse. Specifically, asthma was present in 53.6% of relapsing patients and 56.3% of non-relapsing patients (χ² = 0.1, p = 0.821). NSAID allergy, although rare, was observed in one relapsing patient (100%) and no non-relapsing patients (χ² = 0.8, p = 0.365). Diabetic patients had a relapse rate of 40% compared to 57.6% in non-diabetic patients (χ² = 1.1, p = 0.298). Family history of similar conditions was slightly more common in relapsing patients (63.6%) compared to non-relapsing patients (53.8%), but this difference was not significant (χ² = 0.4, p = 0.546).

Regarding presenting symptoms, certain symptoms showed significant associations with relapse. Facial swelling and decreased vision were strongly associated with relapse, with all patients presenting these symptoms experiencing relapse (χ² = 36.1, p = 0.000*). Anosmia and diplopia were also more common among relapsing patients (69.4% and 80%, respectively). Conversely, epistaxis was more frequent among non-relapsing patients (85.7%).

The duration of the condition and the time from presentation to surgery also influenced relapse rates. Chronic conditions (lasting more than four weeks) had a similar relapse rate (54.8%) to acute conditions (66.7%), though this was not statistically significant (χ² = 0.2, p = 0.685). The time from presentation to surgery was significantly associated with relapse (χ² = 8.3, p = 0.040*). Patients who had surgery within seven months of presentation had higher relapse rates, whereas those who waited longer than seven months had lower relapse rates (40.5%).

The association between pathology-related variables, outcomes, and relapse among the included patients is detailed in Table 5. This analysis reveals various insights into factors contributing to relapse.

**Table 5:** Relapse in association with pathology and outcomes of the condition among the included patients (n=76) (*) indicates a significant p-value < 0.05. HbA1c: hemoglobin A1c; CSF: cerebrospinal fluid

Parameter		Relapse		χ²	p-value
		Non-relapsing	Relapsing		
Surgery complications	No	33 (47.8%)	36 (52.2%)	2.9	0.089
	Yes	1 (14.3%)	6 (85.7%)		
If yes, what are the complications (n=7)	CSF leak	0 (0%)	1 (100%)	2.1	0.71
	Intraoperative bleeding	0 (0%)	1 (100%)		
	Postoperative bleeding	1 (25%)	3 (75%)		
	Synechia	0 (0%)	3 (100%)		
HbA1c	High	6 (54.5%)	5 (45.5%)	0.3	0.557
	Low	16 (44.4%)	20 (55.6%)		
Histopathology	Allergic mucin	10 (50%)	10 (50%)	2.6	0.856
	Allergic nasal polyp	14 (42.4%)	19 (57.6%)		
	Fungal hyphae structure	17 (51.5%)	16 (48.5%)		
	Mucus with inflammatory cells/inflammation	13 (40.6%)	19 (59.4%)		
	Not available	3 (60%)	2 (40%)		
	Positive fungal stain	12 (41.4%)	17 (58.6%)		
Intraoperative culture	Done	15 (36.6%)	26 (63.4%)	2.4	0.122
	Not done	19 (54.3%)	16 (45.7%)		
Intraoperative culture result (n=41)	Negative	7 (63.6%)	4 (36.4%)	4.7	0.029*
	Positive	8 (26.7%)	22 (73.3%)		
Positive culture findings (n=30)	Aspergillus flavus	6 (31.6%)	13 (68.4%)	5.2	0.639
	Aspergillus fumigatus	0 (0%)	1 (100%)		
	Aspergillus niger	0 (0%)	2 (100%)		
	Aspergillus terreus	0 (0%)	1 (100%)		
	*Bipolaris*/*Drechslera*	1 (25%)	3 (75%)		
	Candida albicans	1 (100%)	0 (0%)		
	Dematiaceous fungus *Bipolaris*	0 (0%)	1 (100%)		
Preoperative medications	Antibiotic	4 (36.4%)	7 (63.6%)	3.5	0.83
	Antihistamine	10 (52.6%)	9 (47.4%)		
	Intravenous steroids	0 (0%)	1 (100%)		
	Nasal saline	27 (48.2%)	29 (51.8%)		
	Nasal steroid irrigation (Plumicort)	5 (35.7%)	9 (64.3%)		
	Nasal steroid puffs	23 (44.2%)	29 (55.8%)		
	Oral steroids	15 (46.9%)	17 (53.1%)		
Postoperative medications	Antibiotics	6 (30%)	14 (70%)	7.9	0.343
	Antihistamine	2 (25%)	6 (75%)		
	Nasal saline	28 (42.4%)	38 (57.6%)		
	Nasal steroid irrigation (Plumicort)	20 (50%)	20 (50%)		
	Nasal steroid puffs	9 (37.5%)	15 (62.5%)		
	Oral steroids	16 (43.2%)	21 (56.8%)		
	Xylometazoline	1 (100%)	0 (0%)		
Orbital extension	No	27 (54%)	23 (46%)	5.1	0.024*
	Yes	7 (26.9%)	19 (73.1%)		
Side of orbital extension (n=26)	Both sides	1 (50%)	1 (50%)	2.5	0.28
	Left	2 (14.3%)	12 (85.7%)		
	Right	4 (40%)	6 (60%)		
Skull base affection	No	32 (47.8%)	35 (52.2%)	2.1	0.148
	Yes	2 (22.2%)	7 (77.8%)		
Side of skull base affection	Both	1 (33.3%)	2 (66.7%)	1.3	0.526
	Left	1 (33.3%)	2 (66.7%)		
	Right	0 (0%)	3 (100%)		
Disease involvement	Both sides	15 (42.9%)	20 (57.1%)	2.1	0.346
	Left	7 (35%)	13 (65%)		
	Right	12 (57.1%)	9 (42.9%)		

Surgical complications showed a notable, though not statistically significant, association with relapse. Among patients without surgical complications, 52.2% experienced a relapse, while the relapse rate was markedly higher at 85.7% among those with complications (χ² = 2.9, p = 0.089). Specifically, complications like CSF leak, intraoperative bleeding, postoperative bleeding, and synechia were reported, with postoperative bleeding and synechia occurring exclusively in relapsing patients.

HbA1c levels, categorized as high or low, did not show a significant correlation with relapse. Patients with high HbA1c had a relapse rate of 45.5%, while those with low HbA1c had a slightly higher relapse rate of 55.6% (χ² = 0.3, p = 0.557).

Histopathological findings also did not significantly predict relapse. Patients with allergic mucin, allergic nasal polyps, fungal hyphae structures, and mucus with inflammatory cells had varying relapse rates (50%, 57.6%, 48.5%, and 59.4%, respectively), but these differences were not statistically significant (χ² = 2.6, p = 0.856). Positive fungal stain findings were observed in 58.6% of relapsing patients and 41.4% of non-relapsing patients.

Intraoperative cultures, when performed, showed a significant association with relapse. Among patients with positive intraoperative cultures, 73.3% relapsed compared to 36.4% of those with negative cultures (χ² = 4.7, p = 0.029*). This suggests a strong link between the presence of certain pathogens and the likelihood of relapse. Specific pathogens identified included *Aspergillus *species, with *Aspergillus flavus* being the most common in relapsing patients (68.4%).

Preoperative medication use showed no significant association with relapse. Antibiotic use was associated with a 63.6% relapse rate, antihistamines with 47.4%, and nasal saline with 51.8%. Nasal steroid irrigation and nasal steroid puffs had relapse rates of 64.3% and 55.8%, respectively. The use of oral steroids preoperatively did not significantly alter relapse rates (53.1%).

Postoperative medication use also did not significantly influence relapse rates. Antibiotics were associated with a 70% relapse rate, while antihistamines had a 75% relapse rate. Nasal saline use postoperatively resulted in a 57.6% relapse rate, nasal steroid irrigation (Plumicort) had a 50% relapse rate, and nasal steroid puffs had a 62.5% relapse rate. Oral steroids were used by 56.8% of relapsing patients.

Orbital extension of the disease was significantly associated with relapse, with 73.1% of patients with orbital extension experiencing relapse compared to 46% without orbital extension (χ² = 5.1, p = 0.024*). Among those with orbital extension, involvement of the left side was particularly associated with a higher relapse rate (85.7%).

Skull base affection showed a higher, but not statistically significant, relapse rate. Among patients with skull base affection, 77.8% relapsed compared to 52.2% without skull base affection (χ² = 2.1, p = 0.148). The side of skull base involvement did not significantly influence relapse rates.

Disease involvement in both sides of the sinuses showed a relapse rate of 57.1%, with left-side involvement at 65% and right-side involvement at 42.9%. These differences, however, were not statistically significant (χ² = 2.1, p = 0.346).

## Discussion

AFRS is a complex chronic inflammatory condition characterized by the presence of fungal elements within the sinonasal mucosa [2,3]. It poses significant challenges in management, often requiring surgical intervention such as FESS due to its propensity for recurrence [9,11,12]. Despite advancements in understanding the pathophysiology and treatment of AFRS, the factors contributing to postoperative recurrence remain elusive. Therefore, this study aimed to delve into the demographic, clinical, pathological, and procedural aspects associated with postoperative recurrence in AFRS patients undergoing FESS.

Our study identified several key factors associated with relapse in AFRS patients. These factors include younger age, shorter duration from presentation to surgery, certain presenting symptoms (such as facial swelling and decreased vision), surgical complications, positive intraoperative cultures, and orbital extension. These findings provide valuable insights into the clinical and pathological determinants of relapse in AFRS, which can inform future management approaches.

Our study cohort comprised 76 AFRS patients, predominantly Saudi nationals with a slight female predominance. Comorbidities such as asthma and diabetes mellitus were common among the patients. Postoperative recurrence was observed in 55.3% of cases, with specific factors showing significant associations with relapse. Notably, high HbA1c levels, brain injury, intraoperative bleeding, positive intraoperative cultures, orbital extension, and involvement of the right side in skull base extension were significantly associated with relapse [3,13,16].

Our study found that younger age was significantly associated with relapse in AFRS patients, with relapsing patients having a mean age of 24 ± 9 years compared to 37 ± 17 years in the non-relapsing group (p < 0.001). This age-related difference in relapse rates may be due to the higher immune reactivity typically seen in younger individuals, which could contribute to a more robust inflammatory response to fungal antigens. Additionally, younger patients may have a longer disease duration and more cumulative exposure to allergens, potentially increasing the risk of relapse [13,16].

A shorter interval from presentation to surgery was significantly associated with relapse, with relapsing patients having a mean duration of 229.8 ± 303.3 days compared to 883.9 ± 1145.9 days in non-relapsing patients (p = 0.001). This finding suggests that delaying surgery might allow for better preoperative medical management and optimization, potentially reducing the risk of postoperative relapse. Preoperative medical therapy, including corticosteroids and antifungal treatments, can reduce inflammation and fungal load, thereby enhancing surgical outcomes [3,16].

One of the findings of our study was the association between high HbA1c levels and postoperative recurrence in AFRS patients. This suggests a potential link between glycemic control and disease outcomes, highlighting the importance of managing diabetes mellitus effectively in these patients. The literature supports the role of systemic factors, including diabetes, in influencing the course and severity of chronic rhinosinusitis, of which AFRS is a subtype [13,16,17]. Positive intraoperative cultures were significantly associated with relapse, with 73.3% of relapsing patients having positive cultures compared to 36.4% in the non-relapsing group (p = 0.029). This finding underscores the importance of fungal presence in the pathogenesis of AFRS and highlights the need for targeted antifungal therapy in patients with positive cultures. Antifungal agents, in combination with corticosteroids, can reduce fungal load and inflammation, potentially decreasing the risk of relapse [16-18].

The occurrence of brain injury and intraoperative bleeding in relapsed patients underscores the impact of surgical complications on long-term outcomes. These findings align with previous studies highlighting the significance of meticulous surgical technique and postoperative care in reducing complications and improving patient outcomes post-FESS [18,19]. Strategies aimed at minimizing intraoperative complications, such as thorough preoperative assessment and intraoperative monitoring, may contribute to lowering recurrence rates [13,19].

Positive intraoperative cultures, particularly for *Aspergillus *species, were more prevalent among relapsed patients. This emphasizes the role of fungal colonization in disease persistence and recurrence. Fungal elements not only contribute to the inflammatory cascade in AFRS but also pose challenges in eradicating the disease completely, leading to relapse in susceptible individuals [6,18,20]. Antifungal strategies and targeted interventions may be warranted in cases with persistent fungal colonization to prevent recurrence [5,20].

The presence of orbital and skull base extensions, especially involving the right side, demonstrated significant associations with relapse. Orbital extension was significantly more common in relapsing patients (73.1% vs. 26.9%, p = 0.024). This suggests that the anatomical extent of disease involvement plays a crucial role in predicting postoperative outcomes. Studies have shown that extensive disease involvement, particularly involving critical structures like the orbit and skull base, presents challenges in achieving complete surgical clearance, contributing to higher relapse rates [17,20,21].

Our findings corroborate and extend existing literature on AFRS recurrence post-FESS. Studies identified diabetes as a risk factor for postoperative complications and recurrence in AFRS patients [2-5] and highlighted the impact of fungal colonization on disease persistence and relapse, aligning with our observations regarding positive intraoperative cultures [19,22]. However, our study provides novel insights into the specific associations of surgical complications, glycemic control, and anatomical disease extent with relapse, contributing to a deeper understanding of recurrence mechanisms in AFRS.

The findings from our study have several clinical implications. Firstly, optimizing glycemic control in diabetic AFRS patients may mitigate the risk of postoperative recurrence [8,10]. Secondly, meticulous surgical technique and vigilant postoperative monitoring are crucial in reducing surgical complications and improving long-term outcomes. Thirdly, targeted antifungal strategies and thorough disease evaluation, including assessing the anatomical extent of disease, may aid in personalized management approaches to minimize relapse rates [3,11,13].

The limitations of our study include the small number of patients included. Future studies could involve a larger patient population with a more diverse course of disease. Additionally, there was a lack of previous research on disease course and outcomes.

## Conclusions

In conclusion, our study identified several key factors associated with relapse in AFRS patients, including younger age, shorter time from presentation to surgery, specific presenting symptoms, positive intraoperative cultures, and orbital extension. These findings highlight the need for tailored management strategies to reduce relapse rates, including aggressive and prolonged medical therapy, comprehensive preoperative assessments, meticulous surgical technique, and multidisciplinary care. By addressing these factors, clinicians can improve long-term outcomes for AFRS patients and reduce the burden of this challenging condition. Future research should focus on optimizing preoperative and postoperative management strategies to further reduce relapse rates and improve patient outcomes.
